# Supplementary material for: Rapid Self-Assembly of Metal/Polymer Nanocomposite Particles as Nanoreactors and Their Kinetic Characterization
Source: Nanomaterials (Basel). 2019 Feb 28;9(3):318. doi: 10.3390/nano9030318 (PMC6473589; doi:10.3390/nano9030318)
Supplement: Supplementary file 1 [file nanomaterials-09-00318-s001.pdf]

nanomaterials

Supplementary Materials

Rapid Self-Assembly of Metal/Polymer Nanocomposite Particles as Nanoreactors and their Kinetic Characterization

Andrew Harrison [1], Tien T. Vuong [1], Michael P. Zeevi [1], Benjamin J. Hittel [1], Sungsool Wi [2], and Christina Tang [1,*]

[1] Virginia Commonwealth University, College of Chemical and Life Sciences Engineering, Richmond, VA, 23284-3028, USA
[2] The National High Magnetic Field Laboratory, Florida State University, Tallahassee, FL, 32310, USA
* Correspondence: ctang2@vcu.edu

S1. Methods

S1.1 Kinetic Analysis

Gold nanoreactors (AuNR) (150 uL) produced using FNP were diluted with water (2.275 mL) in a quartz cuvette with a stir bar. The reaction was monitored under stirring with an Ocean Optics FLAME-S-VIS-NIR-ES using an HL-2000-FHSA light source (300-1200 nm), with a CUV-UV cuvette holder (Ocean Optics) placed on a stir plate. The spectrometer was blanked to the reaction mixture. Typically, 4-nitrophenol (25 uL of 0.01M solution) was added to the reaction mixture, and data collection began. After 1 minute, sodium borohydride (50 uL of a 5 M solution) was added to the stirring reaction mixture. Scans were taken every millisecond and averaged over 100 scans with data recorded every quarter-second. The reaction was tracked by monitoring the change in intensity of 4-nitrophenol peak at 425 nm. Absorbance at 425 nm as a function of time was smoothed using a five data point centered moving average to correspond to every second of the experiment. Similar to previous studies [1], the induction time and apparent reaction rate were determined from the absorbance as a function of time.

S1.1.1 Induction Time

Initially, the spectra of the polymer nanoreactor dispersion was recorded as a reference to be subtracted from subsequent spectra as background. The 4-nitrophenol was added resulting in an increase in absorbance at 425 nm. The increase in absorbance due to the addition of 4-nitrophenol occurs within 30 seconds. After 1 minute, the sodium borohydride is added, which results in a further increase in absorbance at 425 nm due to formation of the 4-nitrophenolate ion. The initiation of the reaction corresponding to the start of the induction period was defined as the time at which the absorbance at 425 nm increased to at least 10% of the maximum absorbance of the preceding plateau.

The induction time is characterized by a slow decrease (0.002 Abs/s) in absorbance that is followed by a sharp (> 0.01 Abs/s) decline in absorbance indicating beginning of the reduction reaction, which signifies the end of the induction period. Changes in the slope of the absorbance vs. time was used to quantitatively determine the induction time. Specifically, the absorbance vs. time during the induction time was presumed to be adequately described by a line calculated from fitting the initial ~20% of the induction period. For example, if the induction period lasted roughly 300 seconds based upon visual inspection, the linear fit of the induction time was based on the first 60 seconds of the induction time. The end of the induction time was determined to be the time at which the experimental data deviated from the predicted value for induction time. Specifically, the first occurrence of three consecutive experimental absorbance values that were greater than 5% lower than

the fitted line was classified as the end of the induction period and the beginning of the catalytic reaction.

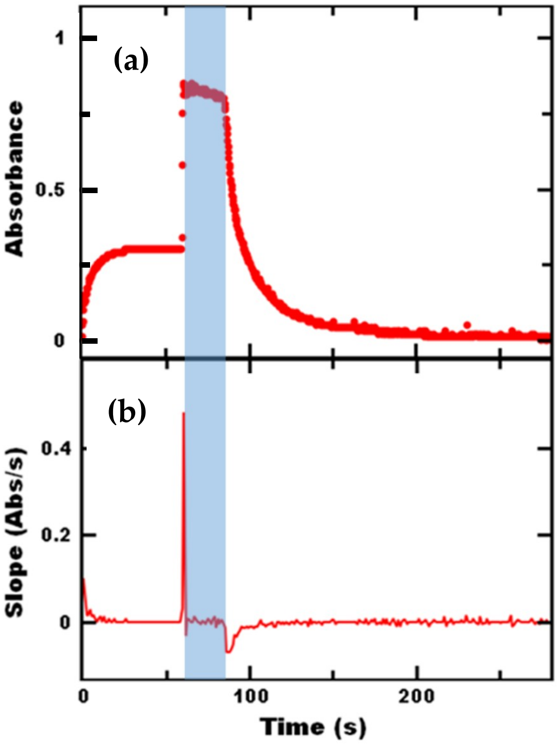

Figure S1: Representative determination of induction time of 4-nitrophenol reduction. (a) Raw reaction data obtained from UV-Vis analysis. Full conversion is achieved when the absorbance reaches zero and occurs within 150 seconds. (b) The 11-point centered change in slope of absorbance over time. The blue shaded region correlates to the induction time.

S1.1.2 Apparent Reaction Rate Constant

For analysis of the reaction rate, the data was normalized to the absorbance value at the end of the induction period. The natural log of the normalized absorbance over time was plotted and regions of two distinct slopes were observed. The first region has been attributed to formation of an intermediate[2]. In order to avoid analysis of the intermediate reaction, the apparent reaction rate was calculated from the second region corresponded to when the normalized absorbance fell below 0.67. Data corresponding to a 15% conversion was analyzed when determining apparent reaction rate. The induction time and apparent reaction rate are reported as the average ± standard deviation of three experimental trials.

Because the reaction was carried out with a large excess of sodium borohydride compared to 4-nitrophenol, the reaction kinetics can be described by pseudo-first-order kinetics. For heterogeneous catalysts, the apparent rate constant is assumed to be proportional to the surface of the catalyst described by[3,4] :

$$-\frac{dc}{dt} = k_{app}c = k_1 S c \quad \text{(S1)}$$

where c is the concentration of 4-nitrophenol at time (t), k_{app} is the apparent rate constant, k_1 is the rate constant normalized to surface area of gold nanoparticles per unit volume of the reaction. Experimentally, k_1 is determined by the change in 4-nitrophenol concentration after the induction

period and the mass of gold measured by ICP (surface area calculated assuming 5 nm spherical particles).

S1.2 NMR-Measurements

Saturation transfer difference (STD) spectroscopy is commonly used in biological molecular interaction studies to analyze ligand-protein interactions [5]. These interactions are probed with and without irradiating the sample by utilizing a very selective pulse, typically a Gaussian pulse with a long pulse duration, at a frequency identical to the resonance frequency the host molecule that is in close spatial proximity to the ligand molecule of interest. Moreover, due to spin diffusion, the signals from the ligands associated with the host molecule will be attenuated as well. A peak subtraction is pursed between the spectra obtained with and without the selective irradiation. The interacting ligands are then probed from the difference spectrum.

To evaluate effective transport of the 4-nitrophenol, ^{1}H-NMR spectroscopy and pulsed field gradient (PFG) NMR were performed using a Bruker Avance II 800 MHz NMR with a 5-mm coil 1H-X-Y TBI solution state cryo-NMR probe operating at a narrow-bore and 18.8 T magnet with 50 G/cm gradient along the z-direction. Nanoreactor samples in D_2O and 4-nitrophenol were equilibrated for 100 minutes prior to the PFG-NMR experiment. The self-diffusion coefficients *D* were determined from the proton spin-echo intensities measured as a function of gradient pulse strength using a standard DOSY sequence:

$$\boldsymbol{ln(I/I_0) = -D\gamma^2 g^2 \delta^2 \left(\Delta - \frac{\delta}{3} - \frac{\tau}{2}\right)} \quad \text{(S2)}$$

where I_0 is the signal amplitude after the PFG pulse sequence with minimal gradients applied, $\boldsymbol{\gamma}$ the gyromagnetic ratio, *g* the gradient strength applied, $\boldsymbol{\delta}$ is the gradient pulse duration (1 ms), $\boldsymbol{\Delta}$ is the diffusion time, and *D* the self-diffusion coefficient of the mobile species. Parameters employed in our experiments were: g = 46.99 G/cm; $\boldsymbol{\Delta}$ = 0.1 s; $\boldsymbol{\delta}$ = 1 ms; $\boldsymbol{\tau}$ = 514 μs. A standard solution (1% H_2O/99% D_2O, doped with 0.1 mg $GdCl_3$) with established diffusion coefficient (1.872×10^{-9} m^2/s at 298 K) was used for calibration.

Combined saturation transfer difference (STD) spectroscopy and PFG NMR were used to isolate the solute molecules associated with the nanoreactors. Selective saturation of the reactor was achieved by a train of Gaussian-shaped pulses of 30 ms, saturating a bandwidth of about 20 Hz, at 6.88 ppm (where the nanoreactor has signals, but the solute does not) for a saturation time of approximately 3 s to ensure full saturation of the nanoreactor. A reference spectrum and PFG spectra were obtained by irradiating at 0 ppm spectrum. A difference spectra between 0 ppm and 6.8 ppm was obtained (0 ppm spectrum –6. 88 ppm spectrum) to analyze the solute molecules that are within the reactor. Using the PFG spectra, the solute peak intensity as a function of gradient strength was plotted and the diffusion coefficient was determined from the slope of the linear fit.

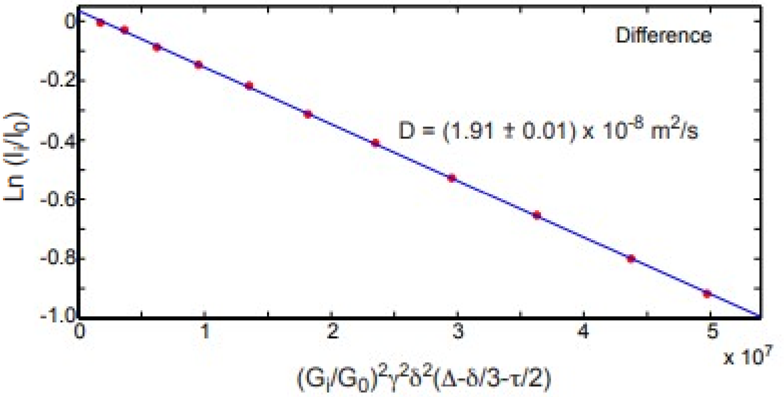

Figure S2: Results of saturation transfer differentiated PFG-NMR of 4-nitrophenol in a solution of polystyrene nanoreactors. The blue line corresponds to the curve fit. The difference spectra correspond to the signal from the 4-nitrophenol in closest proximity to polystyrene (within the nanoreactors), which we interpret as an effective diffusion coefficient of 4-nitrophenol within the nanoreactors of 1.9 x 10^{-8} m^2/s.

S1.3 Langmuir Hinshelwood Kinetic Analysis

The Langmuir Hinshelwood Kinetic Analysis is influenced by the data available at the end of the induction time and start of the reaction. Therefore, we used a more precise method to define the induction time. Prior to the addition of 4-nitrophenol or sodium borohydride, a background spectra of the polymer nanoreactors was taken in order to adjust the reaction data. With the addition of 4-nitrophenol, the absorbance at 425 nm could be seen to rise from the initial absorbance of zero. A plateau in absorbance occurs within 30 seconds after the addition of 4-nitrophenol, which is disturbed by the addition of sodium borohydride. The resulting pH change and presence of the 4-nitrophenolate ion spikes the measured absorbance 10% over the maximum absorbance of the preceding plateau, which begins the period of induction time. A slow decrease (0.002 Abs/s) in absorbance is then noted over the following induction period, after which a sharp decline in absorbance indicates the beginning of the reduction reaction and the termination of the induction period.

The slope of absorbance was calculated over a centered 11-point data range. Noise in the calculated slope was determined to be 10% of the maximum peak absorbance change prior to the beginning of the induction time. The first negative slope change that had a magnitude greater than the defined noise and was consistent over the following two data points was considered the beginning of the reduction reaction and the termination of the induction time. All of the data was then normalized to the absorbance value at the time point, marking the end of the induction period. The reaction data immediately following the induction period was fit to the Langmuir Hinshelwood model.

The reduction of 4-nitrophenol catalyzed by metal nanoparticles model is fully described considering two intermediates: 4-nitrosophenol and 4-hydroxylaminophenol. Since 4-hydroxylaminophenol is the first stable intermediate, there are three compounds that adsorb and desorb during the reaction cycle (reactant, intermediate, and product) and compete for a fixed number of sites on the surface of the nanoparticle catalysts. The surface coverage is modeled as a Langmuir-Freundlich isotherm following previous reports [1]. The reaction is modelled using two steps: (A) the reduction of 4-nitrophenol to 4-hydroxylaminophenol and (B) the reduction of 4-hydroxylaminophenol to 4-aminophenol and step B is rate limiting. The resulting coupled rate equations are solved numerically to fully model the concentration of 4-nitrophenol as a function of time to fit experimental data (normalized after the induction time for conversions up to 30%) as

described previously [1]. The model and experimental data are plotted in Figure S3 with good agreement. The full fit parameters are provided in Table S1.

Table S1: Langmuir-Hinshelwood Fitting Parameters [2].

K_{nip} (L/mol)	K_{bh} (L/mol)	K_{hx} (L/mol)	S (m^2/L)	C_{bh} (mol/L)	C_{nip} (mol/L)	n
4600	62	175000	0.084	0.1	0.0001	0.5

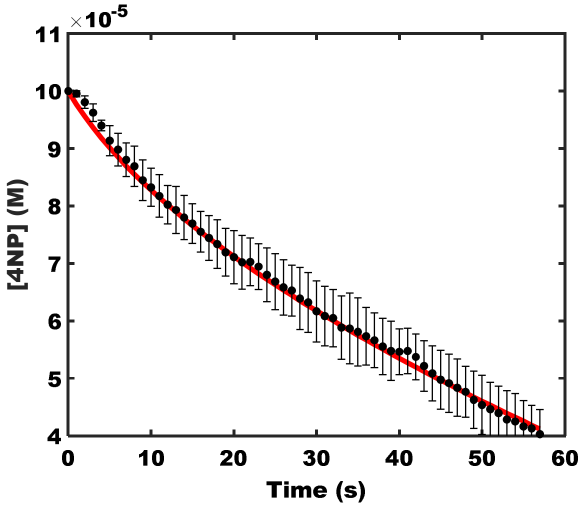

Figure S3: Langmuir-Hinshelwood model fit of experimental data for polystyrene nanoreactors. The black circles correspond to experimental data points and the red line represents the fitted curve.

Since the data is well described by the model, the assumptions of first-order rate kinetics, without mass transfer limitations, appear valid within the polymer microenvironment. For fitting, we assume the initial concentrations of the reagents are the same as the bulk. Given the high solubility of borohydride in water compared to organic solvents, the concentration of sodium borohydride of local microenvironment may be lower than the bulk, which may lead to underestimation of the rate constants.

S1.4 Leaching Studies

To confirm that the observed catalytic activity is associated with the nanoreactors, we compared the nanoreactors to the nanoparticles that were added to polystyrene nanoparticles made via FNP under rigorous stirring with multiple reuses. For reuse following the reaction, nanoreactors were recovered using an Amicon Ultra 2 mL 50K centrifugal filter according to manufacturer's instructions. The filtrate was collected and the retentate containing nanoreactors was diluted with DI water to the original volume. The recovered nanoreactors were diluted with water to their initial nominal concentration. Finally, the 4-nitrophenol reduction was performed again. This process was carried out three times. For analysis of catalyst leaching, the cumulative filtrate collected for a single sample over multiple recycling steps was dissolved in aqua regia and analyzed with ICP-OES.

In both cases, there was an observed decrease in K_{app} after reuse. The gold nanoparticles added to the polymer nanoparticles lost all activity after the first recycling step (Figure S4a). For the gold nanoparticles added to polymer nanoparticles, no catalytic activity was observed after the first recycle whereas the nanoreactors retain activity after three recycles (Figure S4a), indicating that the retained activity can be attributed to the nanoreactors.

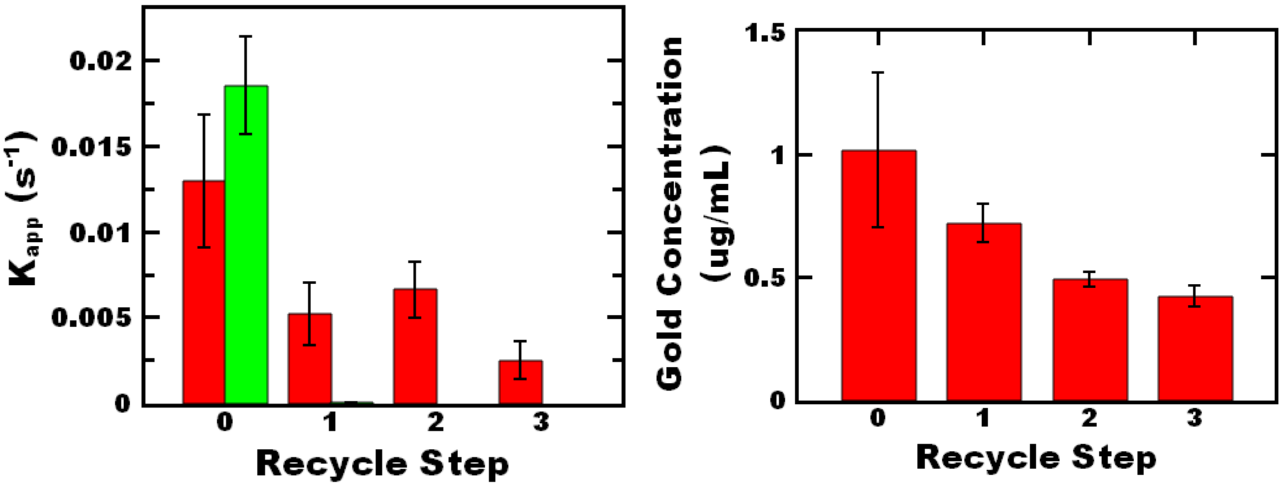

(a) (b)

Figure S4: Leaching studies of polymer nanoreactors and polystyrene nanoparticles with gold nanoparticles added to solution. (a) The apparent rate constants of polymer nanoreactors (red bars) and polystyrene nanoparticles with added gold nanoparticles (green bars) after subsequent recycling steps. (b) The gold concentration of the reaction solution at each recycling step for polymer nanoreactors. No activity is seen from the polystyrene nanoparticles with added gold nanoparticles after 1 recycling step.

The decrease in k_1 after three recycles suggests leaching from the gold nanoreactor does occur with multiple reuses. Performing ICP on the filtrate, there was ~30% reduction in gold content with each recycle step; thus, the loss in activity corresponds to loss of gold. Since 70% retention of nanoparticles has been reported using centrifugal based separations [6], the loss of gold can be attributed, in part, to the loss of nanoreactors.

Table S2: Reaction rate constant per catalyst surface area in the reaction solution after nanoreactor recycling.

Recycle Step	k_1 (L m^{-2} s^{-1})
0	0.4139 ± 0.0952
1	0.2246 ± 0.0901
2	0.4419 ± 0.1266
3	0.1984 ± 0.0632

S2. Results and Discussion

S2.1 Nanoreactor Formulation and Characterization

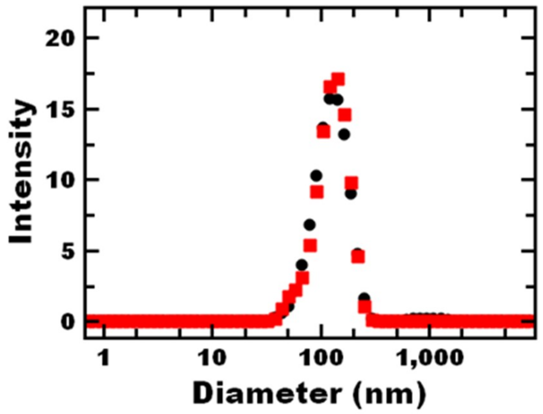

Figure S5: Nanoreactor stability at room temperature. No significant change in hydrodynamic size or PDI was observed by DLS after 8 weeks of storage (red squares) compared to the initial size distribution (black circles).

In this system, the nanoreactor size could be tuned from 110 nm to 170 nm by increasing the block copolymer concentration while holding the core concentration constant (Supporting Information, Figure S3). As expected, the overall nanoreactor size decreased with increasing block copolymer concentration. This trend has been attributed to the relative change in core volume relative to the block copolymer surface area [7] Since nanoreactor formation is arrested by adsorption of the block copolymer to the growing core, increasing the block copolymer concentration results in faster nanoreactor formation and smaller particles due to the reduced time allowed for growth. However, in this approach to tune particle size, the nanoreactor size and gold loading (mass of gold per total mass of nanoreactor) are affected.

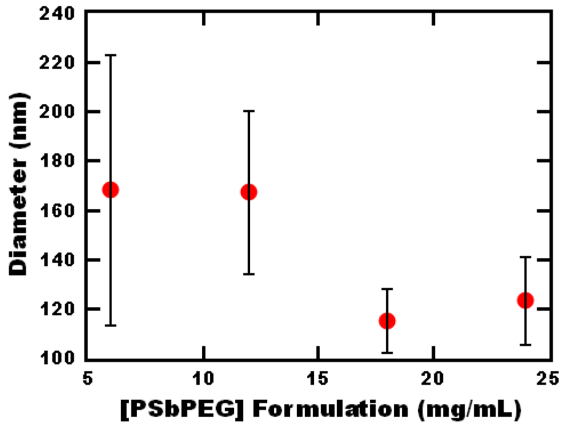

Figure S6. Effect of block copolymer concentration on nanoreactor size. Increasing the block copolymer concentration resulted in a decrease in nanoreactor size and gold loading.

S2.2 Kinetic Analysis

S2.2.1 Induction Time

Since the mass transfer is also related to concentration, we examined the effect of reagent (4-nitrophenol and sodium borohydride at a constant ratio) concentration on induction time. At the standard reaction conditions (0.1 M $NaBH_4$), induction times ~100 seconds (20-fold longer than citrate-stabilized gold nanoparticles) were observed when the equilibration time between adding the sodium borohydride followed by the 4-nitrophenol was less than 3 minute (Figure 7). At a lower concentration (0.01 M $NaBH_4$), a similar induction time was observed for equilibration times less than 10 minutes. In contrast, at higher concentration (0.2 M $NaBH_4$), the induction time was ~5 seconds (comparable to citrate stabilized gold nanoparticles) for equilibration times of at least one minute. These result further suggest the long induction time of the nanoreactors relative to citrate stabilized gold nanoparticles can be attributed to diffusion of the borohydride.

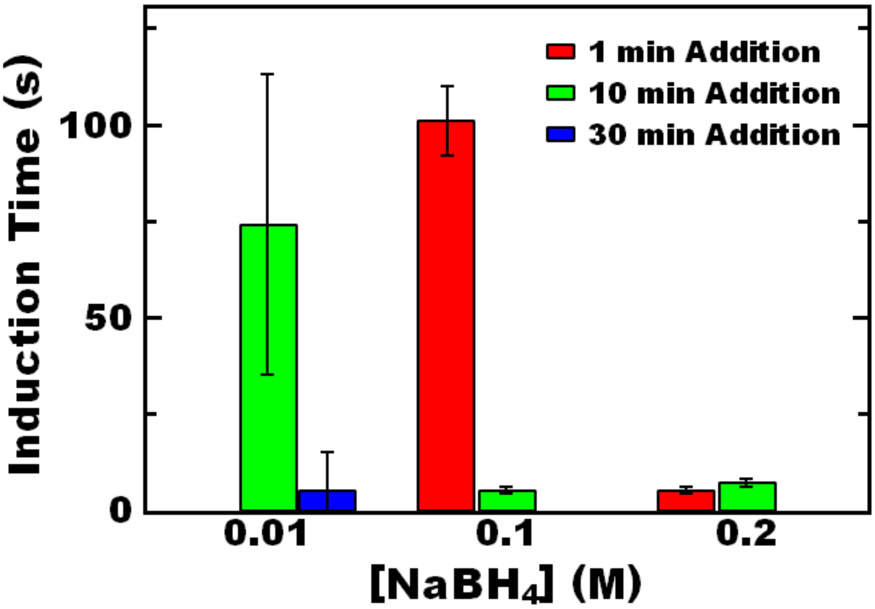

Figure S7: The effect of 4-nitrophenol interval of addition (reverse addition sequence) on the induction time present in the reduction of the 4-nitrophenol at varying concentration of reagent. As reagent concentration increases, the length of addition interval necessary to achieve the minimum induction time of roughly 5 seconds (Figure 7) decreases.

S2.2.2 Scaling Analysis

A complementary scaling analysis approach is to consider the bimolecular reaction between 4-nitrophenol and nanoparticle catalyst using the Smoluchowski diffusion limited reaction model [8,9]. In the limit of slow diffusion, the bimolecular rate constant, k_{bm}, is

$$k_{bm} = 4\pi r D \tag{S3}$$

The value of the k_{bm} can be compared to the experimentally determined 2nd order constants as an indication of mass transfer limitations. Since the concentration of the nanoparticle is constant over the course of the reaction, the observed disappearance of 4-nitrophenol follows a pseudo-first-order reaction with rate constant k_{app}, i.e.,

$$\frac{d[4NP]}{dt} = k[4NP][Au] = k_{app}[4NP] \tag{S4}$$

where [4NP] and [Au] are 4-nitrophenol concentration and gold nanoparticle catalyst concentration respectively, k is the 2nd order rate constant and k_{app} is the pseudo-first-order rate constant and

$$k_{app} = k[Au] \quad \text{(S5)}$$

Therefore, the 2nd order rate constant, k, can be determined from measuring the k_{app} as a function of catalyst concentration [8,9]. An experimentally determined k value approaching k_{bm} would suggest a diffusion limitation.

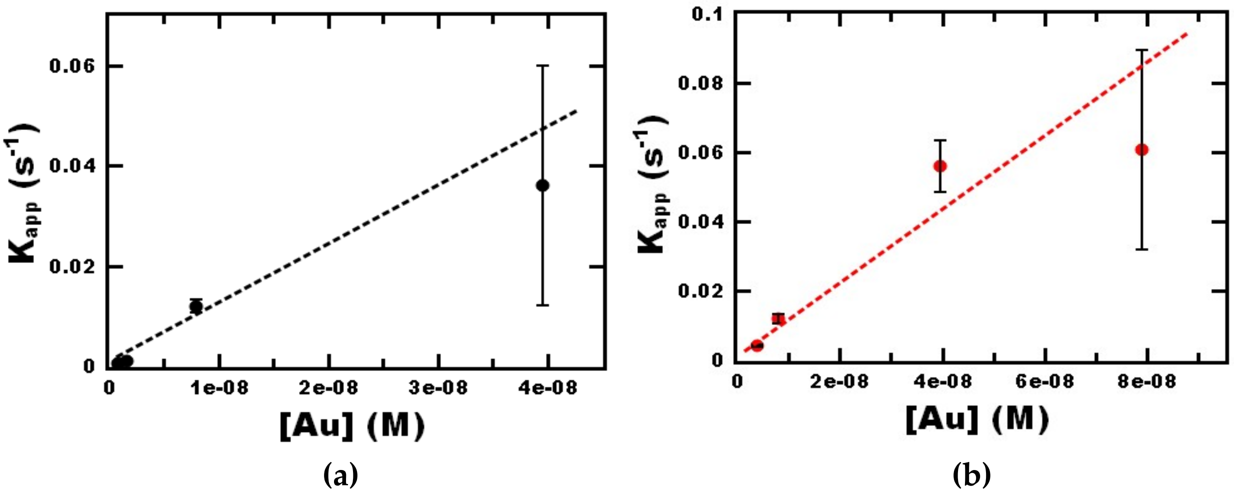

Figure S8: The effect of gold concentration on the reaction rate kinetics of 4-nitrophenol reduction by varying (a) the nanoreactor concentration to probe external mass transfer and (b) varying the nanoreactor loading to probe internal mass transfer.

When the nanoreactor concentration or the gold loading was increased, k_{app} increased; the 2nd order rate constant was on the order of 10^6 $M^{-1}s^{-1}$. These values are much lower than the k_{bm} ~ 10^8 $M^{-1}s^{-1}$ indicating that neither internal nor external diffusion from the bulk solution to the nanoreactor limit the apparent reaction kinetics.

References

1. Momot, K. I.; Kuchel, P. W. Pulsed Field Gradient Nuclear Magnetic Resonance as a Tool for Studying Drug Delivery Systems. *Concepts Magn. Reson.* **2003**, *19A*, 51–64. doi:10.1002/cmr.a.10092.
2. Gu, S.; Wunder, S.; Lu, Y.; Ballauff, M.; Fenger, R.; Rademann, K.; Jaquet, B.; Zaccone, A. Kinetic Analysis of the Catalytic Reduction of 4-Nitrophenol by Metallic Nanoparticles. *J. Phys. Chem. C* **2014**, *118*, 18618–18625. doi:10.1021/jp5060606.
3. Gu, S.; Kaiser, J.; Marzun, G.; Ott, A.; Lu, Y.; Ballauff, M.; Zaccone, A.; Barcikowski, S.; Wagener, P. Ligand-Free Gold Nanoparticles as a Reference Material for Kinetic Modelling of Catalytic Reduction of 4-Nitrophenol. *Catal. Letters* **2015**, *145*, 1105–1112. doi:10.1007/s10562-015-1514-7.
4. Fenger, R.; Fertitta, E.; Kirmse, H.; Thünemann, A. F.; Rademann, K. Size Dependent Catalysis with CTAB-Stabilized Gold Nanoparticles. *Phys. Chem. Chem. Phys.* **2012**, *14*, 9343–9349. doi:10.1039/c2cp40792b.
5. Viegas, A.; Manso, J.; Nobrega, F. L.; Cabrita, E. J. Saturation-Transfer Difference (STD) NMR: A Simple and Fast Method for Ligand Screening and Characterization of Protein Binding. *J. Chem. Educ.* **2011**, *88*, 990–994. doi:10.1021/ed101169t.
6. Zhang, M.; Liu, L.; Wu, C.; Fu, G.; Zhao, H.; He, B. Synthesis, Characterization and Application of Well-Defined Environmentally Responsive Polymer Brushes on the Surface of Colloid Particles. *Polymer (Guildf).* **2007**, *48*, 1989–1997. doi:10.1016/j.polymer.2007.01.069.

7. Pagels, R. F.; Edelstein, J.; Tang, C.; Prud'homme, R. K. Controlling and Predicting Nanoparticle Formation by Block Copolymer Directed Rapid Precipitations. *Nano Lett.* **2018**, *18*, 1139–1144. doi:10.1021/acs.nanolett.7b04674.
8. Chang, Y. C.; Chen, D. H. Catalytic Reduction of 4-Nitrophenol by Magnetically Recoverable Au Nanocatalyst. *J. Hazard. Mater.* **2009**, *165*, 664–669. doi:10.1016/j.jhazmat.2008.10.034.
9. Ncube, P.; Bingwa, N.; Baloyi, H.; Meijboom, R. Catalytic Activity of Palladium and Gold Dendrimer-Encapsulated Nanoparticles for Methylene Blue Reduction: A Kinetic Analysis. *Appl. Catal. A Gen.* **2015**, *495*, 63–71. doi:10.1016/j.apcata.2015.01.033.

© 2019 by the authors. Submitted for possible open access publication under the terms and conditions of the Creative Commons Attribution (CC BY) license (http://creativecommons.org/licenses/by/4.0/).
